# Development and Validation of Liquid Chromatography-Tandem Mass Spectrometry Methods for the Quantification of Cefquinome, Ceftiofur, and Desfuroylceftiofuracetamide in Porcine Feces with Emphasis on Analyte Stability

**DOI:** 10.3390/molecules26154598

**Published:** 2021-07-29

**Authors:** Sofie Rutjens, Siska Croubels, Siegrid De Baere, Mathias Devreese

**Affiliations:** Department of Pharmacology, Toxicology and Biochemistry, Faculty of Veterinary Medicine, Ghent University, 9820 Merelbeke, Belgium; Sofie.Rutjens@UGent.be (S.R.); Siska.Croubels@UGent.be (S.C.); Siegrid.DeBaere@UGent.be (S.D.B.)

**Keywords:** cefquinome, ceftiofur, desfuroylceftiofuracetamide, (U)HPLC-MS/MS, porcine feces, method validation

## Abstract

Cefquinome and ceftiofur are β-lactam antibiotics used for the treatment of bacterial infections in swine. Although these antimicrobials are administered intramuscularly, the exposure of the gut microbiota to these cephalosporins is not well described. This exposure can contribute to the emergence and spread of antimicrobials in the environment and to the possible spread of antimicrobial resistance genes. To assess the impact of drug administration on the intestinal excretion of these antimicrobials it is essential to measure the amounts of native compound and metabolites in feces. Two (ultra)-high-performance liquid chromatography-tandem mass spectrometry ((U)HPLC–MS/MS) methods were developed and validated, one for the determination of cefquinome and ceftiofur and the other for the determination of ceftiofur residues, measured as desfuroylceftiofuracetamide, in porcine feces. The matrix-based calibration curve was linear from 5 ng g^−1^ to 1000 ng g^−1^ for cefquinome (correlation coefficient (r) = 0.9990 ± 0.0007; goodness of fit (gof) = 3.70 ± 1.43) and ceftiofur (r = 0.9979 ± 0.0009; gof = 5.51 ± 1.14) and quadratic from 30 ng g^−1^ to 2000 ng g^−1^ for desfuroylceftiofuracetamide (r = 0.9960 ± 0.0020; gof = 7.31 ± 1.76). The within-day and between-day precision and accuracy fell within the specified ranges. Since β-lactam antibiotics are known to be unstable in feces, additional experiments were conducted to adjust the sampling protocol in order to minimize the impact of the matrix constituents on the stability of the analytes. Immediately after sampling, 500 µL of an 8 µg mL^−1^ tazobactam solution in water was added to 0.5 g feces, to reduce the degradation in matrix.

## 1. Introduction

Cefquinome ((6*R*,7*R*)-7-[[(2Z)-2-(2-amino-1,3-thiazol-4-yl)-2-methoxyiminoacetyl]amino]-8-oxo-3-(5,6,7,8-tetrahydroquinolin-1-ium-1-ylmethyl)-5-thia-1-azabicyclo [4.2.0]oct-2-ene-2-carboxylate) and ceftiofur ((6*R*,7*R*)-7-[[(2*Z*)-2-(2-amino-1,3-thiazol-4-yl)-2-methoxyiminoacetyl]amino]-3-(furan-2-carbonylsulfanylmethyl)-8-oxo-5-thia-1-azabicyclo[4.2.0]oct-2-ene-2-carboxylic acid), are both β-lactam compounds structurally belonging to the cephalosporins. In veterinary medicine both compounds are used for the parenteral treatment of bacterial infections in livestock caused by various Gram-positive and Gram-negative organisms [1,2,3,4,5,6,7].

After absorption, the thioester bond of ceftiofur is rapidly cleaved (see Figure S1) with the formation of an active metabolite, desfuroylceftiofur, as well as furoic acid [4]. The sulfhydryl moiety of desfuroylceftiofur can form reversible, covalent bindings to different constituents, resulting in the presence of a wide range of metabolites (desfuroylceftiofur cysteine disulfide, desfuroylceftiofur glutathione disulfide, desfuroylceftiofur dimer, desfuroylceftiofur protein). Since these metabolites still contain the β-lactam ring, they have the potential to be microbiologically active [8].

Furthermore, these antimicrobials and their metabolites are excreted either via urine or feces. For cefquinome and ceftiofur, excretion via urine is the main excretion pathway in pigs. Only about 7% of the administered dose of ^14^C-cefquinome and about 10% of the administered dose of ^14^C-ceftiofur gets excreted via the feces [9,10]. Although this fraction is limited, this fecal excretion exposes the gut bacteria to these residues. Several studies have indicated that this exposure leads to the development of resistant bacteria, confirming the hypothesis that these residues are still antimicrobiologically active [11,12,13,14,15]. After excretion, both these antibiotic residues and resistant bacteria can spread in the environment, becoming a threat to human health [16,17,18,19]. Determining the concentration of native compounds and antimicrobiologically active metabolites in feces will provide information on the possible effect of parenteral administration on the exposure of the gut bacteria and on the environmental spread of cefquinome, ceftiofur and ceftiofur metabolites [20,21].

However, the chemical analysis of β-lactam antibiotics in fecal samples is not straightforward and includes several challenges. Pig feces, and feces in general, is considered to be a complex matrix, resulting in the need for advanced extraction protocols [22,23]. Furthermore, it has been reported that the gut bacteria can hydrolyze the β-lactam ring structure, and thus an adequate post-sampling protocol is necessary to obtain accurate results [19].

Only a handful of methods have been published for the determination of cefquinome and ceftiofur in manure [24,25,26]. Since these methods only measure ceftiofur, which gets rapidly metabolized into its active in vivo metabolite, desfuroylceftiofur, the recovery of ceftiofur, as such, in manure, and thus in feces, will be minimal. This results in an underestimation of the gastro-intestinal exposure to antimicrobially active compounds. Furthermore, these methods target manure or hen droppings, which have a different constitution than pig feces and, therefore, require a different sample extraction. For the analysis of ceftiofur in combination with its metabolites, several methods for matrices different from feces are published, which derivatize ceftiofur and all its metabolites to desfuroylceftiofuracetamide (DFCA) [8,27,28,29,30]. However, to the authors’ knowledge, there is no analytical method published that determines DFCA in fecal samples.

The objective of this study was to develop and validate two (ultra)-high-performance liquid chromatography-tandem mass spectrometry ((U)HPLC-MS/MS) methods, one for the quantification of cefquinome and ceftiofur, and the other for the determination of ceftiofur residues, measured as DFCA, in porcine feces. Several additional stability studies were conducted to determine a suitable sampling protocol, in order to minimize post-sampling degradation. To show the applicability of the method, the method was tested by analyzing fecal samples of healthy pigs after administration of cefquinome and ceftiofur.

## 2. Results

### 2.1. Mass Spectrometry

The structures of cefquinome, ceftiofur and DFCA and their MS and MS/MS traces are shown in Figure S1. The MS/MS parameters of cefquinome and ceftiofur were obtained by direct infusion of a working solution of 1 µg mL^−1^. Those of DFCA were obtained by direct infusion of a derivatized ceftiofur working solution of 1 µg mL^−1^. The most abundant mass peaks in the MS mode for cefquinome, cefquinome-d7, ceftiofur, ceftiofur-d3, DFCA and DFCA-d3 were ions [M + H]^+^ at *m*/*z* 529.0, 536.3, 524.0, 527.0, 487.2 and 490.2, respectively. After optimizing the MS parameters, the precursor ions were fragmented, and cone voltages and collision energies were selected (Table 1). The fragmentation pattern of ceftiofur was in accordance with literature [31]. The most abundant product ion was chosen as the quantifier ion, whereas the second most intense product was chosen as qualifier ion.

### 2.2. Chromatography

The best results were obtained using a Zorbax Eclipse plus C18 column 100 × 2.1 mm i.d. The selected mobile phase was H_2_O containing 0.1% (*v*/*v*) formic acid and 2 mM ammonium formate as mobile phase A and acetonitrile (ACN) containing 0.1% (*v*/*v*) formic acid as mobile phase B.

For the analysis of DFCA, an unpublished in-house validated method for the detection of DFCA in muscle was used. In this method, good results were obtained using an Acquity UPLC BEH C18 column 50 *×* 2.1 mm i.d. combined with a guard column of the same type. The mobile phases for chromatographic separation consisted of 0.005% (*v*/*v*) formic acid in H_2_O (A) and acetonitrile (B). Preliminary experiments with these chromatographic settings showed good results on extracted spiked fecal samples, so no adaptations were made.

Retention times and a representative chromatogram of spiked blank fecal samples, incurred samples and a blank fecal sample can be found in Figure S2.

### 2.3. Analysis of Biological Samples

The intramuscular (IM) injection of Excenel flow^®^ resulted in a concentration of 85.4 ng g^−1^ ceftiofur in feces at 2 h after injection and 30.5 ng g^−1^ DFCA at 10 h after injection. The IM injection of Ceffect^®^ resulted in a concentration of 7.1 ng g^−1^ cefquinome in feces at 2 h after injection. The chromatograms of these samples can be found in Figure S2.

### 2.4. Stability Experiments

#### 2.4.1. Freeze-Thaw, Matrix and Extract Storage Stability

The results of the freeze-thaw, matrix and extract storage stability evaluation are summarized in Table S1. For cefquinome and ceftiofur, the acceptability ranges were met for the storage stability in matrix and extract. For both analytes, the acceptability ranges were not met for the freeze-thaw stability at Limit Of Quantification (LOQ) level. For ceftiofur, measured as DFCA, the acceptability ranges were met for the freeze-thaw and matrix stability at the specified levels. The acceptability ranges were not met for the stability of DFCA in extracted samples at the LOQ level. For desfuroylceftiofur, measured as DFCA, the acceptability ranges were met for both freeze-thaw and matrix stability. Since desfuroylceftiofur is derivatized into DFCA, the extract stability of DFCA was only tested once during the stability tests of ceftiofur combined with the derivatization method.

#### 2.4.2. Bench Top Stability

**Figure 1:**
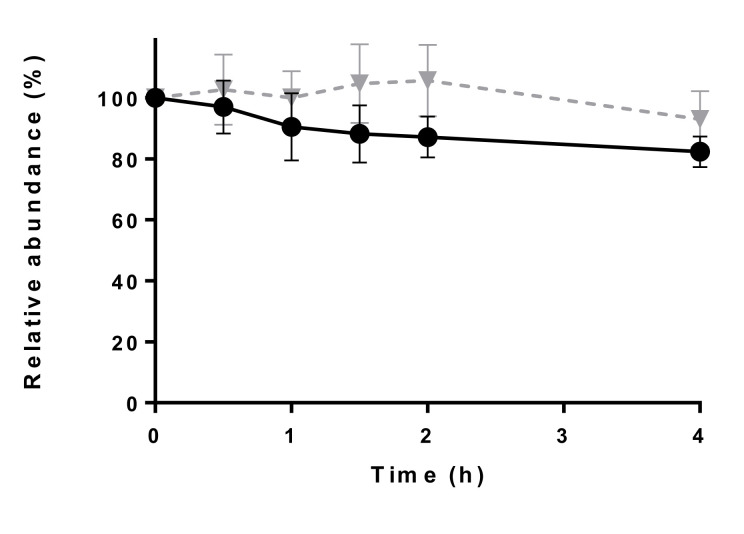
Degradation of a 1 µg mL^−1^ cefquinome solution in ultra high-performance liquid chromatography-water (UHPLC-water) (-▼-) and a 1 µg mL^−1^ ceftiofur (●) working solution in dimethyl sulfoxide/UHPLC-water (1/1000; *v*/*v*) (mean ± standard deviation) at room temperature (21 ± 2 °C; *n* = 3).

The stability of the working solutions at room temperature (21 ± 2 °C) of both cefquinome and ceftiofur was evaluated to ensure that the degradation in matrix was only due to the matrix constituents and not due to the instability of the analytes in solution. Figure 1 shows the change in absolute peak area with time point 0 being equal to 100%. The difference between the absolute area at time point 0 and the absolute area at the other time points (in %) fell within the −20 to +10% acceptability range. Therefore, it can be concluded that the stability of the analytes in working solution is acceptable.

**Figure 2:**
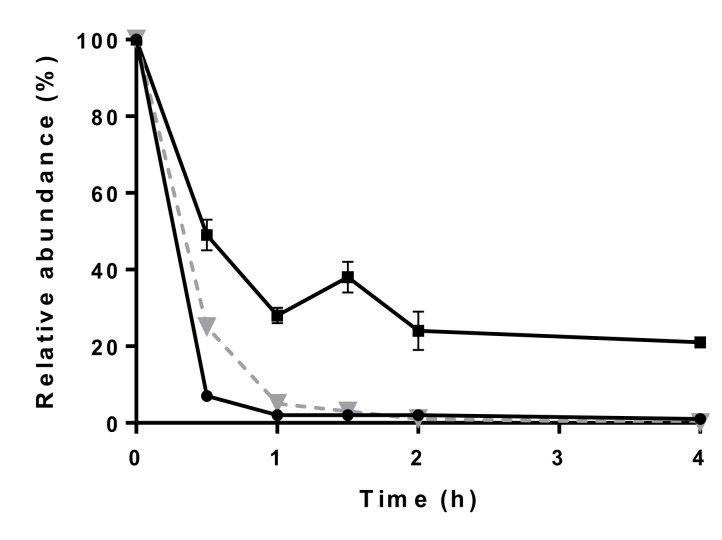
Degradation at room temperature (21 ± 2 °C) over time of cefquinome (-▼-), ceftiofur (●) and desfuroylceftiofur (■) in a 1000 ng g^−1^ spiked fecal sample (mean ± standard deviation, *n* = 3, per time point).

The stability of cephalosporins in feces is known to be limited. Several commensal bacteria are capable of hydrolyzing the β-lactam ring, resulting in the loss of their antibacterial activity [4]. To ensure the most accurate post-sampling concentration, it is interesting to assess the rate of degradation and adjust the sampling protocol accordingly. The decrease in concentration of ceftiofur is due to a combination of hydrolysis of the β-lactam ring and cleavage of the thioester bond, whilst the decrease in concentration of desfuroylceftiofur is caused exclusively by the hydrolysis of the β-lactam ring. The decrease in concentration of cefquinome, ceftiofur and desfuroylceftiofur is depicted in Figure 2.

**Figure 3:**
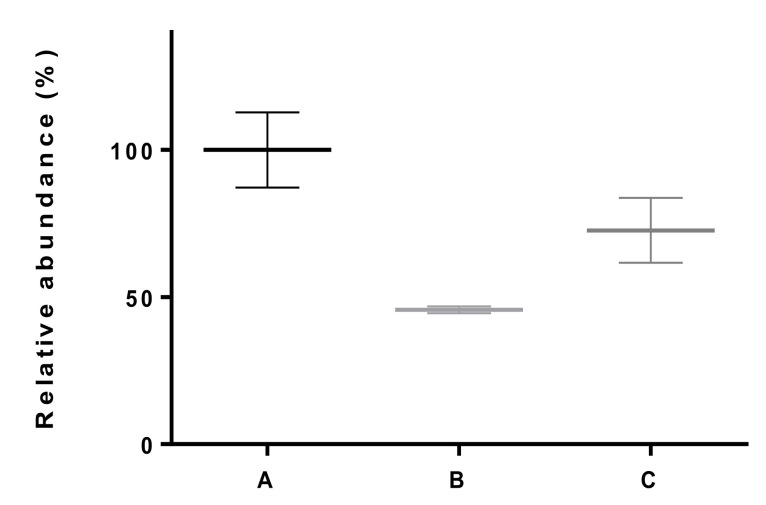
Effect of snap-freezing with liquid nitrogen on the DFCA concentration in a 1000 ng g^−1^ spiked fecal sample. Comparison of immediately extracted and analyzed samples (A, *n* = 3, mean% = 100 ± standard deviation)), with snap-frozen and thawed samples (B, *n* = 3, mean% = 47 ± standard deviation)) and with snap-frozen and unthawed samples (C, *n* = 3, mean% = 72 ± standard deviation)).

The effect of snap-freezing in liquid nitrogen on the ceftiofur concentration in fecal samples (measured as DFCA) was examined. Figure 3 shows the results of snap-freezing spiked samples combined with or without thawing prior to analysis, in comparison to immediately analyzed samples.

A clear deterioration of DFCA (>50%) can be observed for the samples that were snap-frozen and thawed prior to analysis (Figure 3). This deterioration can also be seen for the samples that were not thawed (>25%, Figure 3), albeit to a lesser extent. Since the mean of the samples in protocol C fell outside the −20 to +10% acceptability range, this sampling method was discontinued.

**Figure 4:**
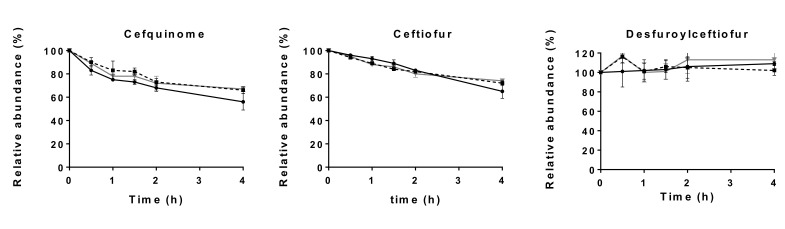
Degradation at room temperature (21 ± 2 °C) over time of cefquinome, ceftiofur and desfuroylceftiofur in a 1000 ng g^−1^ spiked fecal sample (*n* = 3, per time point) in the presence of a 4 µg mL^−1^ (●), 8 µg mL^−1^ (-■-) or 16 µg mL^−1^ (▼) solution of tazobactam (mean ± standard deviation).

Hence, another procedure to ensure the post-sampling stability of the analytes was tested. To spiked blank fecal samples, tazobactam, a β-lactamase inhibitor, was added at different concentrations. Figure 4 shows the degradation at room temperature of cefquinome, ceftiofur and desfuroylceftiofur in spiked fecal samples at a concentration of 1000 ng g^−1^ after addition of 500 µL of either a 4 µg mL^−1^, 8 µg mL^−1^ or 16 µg mL^−1^ solution of tazobactam in ultrapure water.

The results of this stability experiment indicate that there is still degradation of cefquinome and ceftiofur in fecal samples, regardless of the addition of a β-lactamase inhibitor. However, a much slower degradation is seen than without the addition of tazobactam. Desfuroylceftiofur is not subject to this degradation. Since without tazobactam degradation of ceftiofur and cefquinome at room temperature is unavoidable, it was decided to add 500 µL of a 8 µg mL^−1^ tazobactam solution to 0.5 g feces immediately after sampling and to store the samples at ≤−70 °C within 30 min post sampling.

### 2.5. Method Validation

#### 2.5.1. Linearity, Limit of Quantification and Limit of Detection

For the matrix-matched calibration curves, good linearity was observed up to 2000 ng g^−1^ for DFCA, and up to 1000 ng g^−1^ for ceftiofur and cefquinome. Table 2 shows the goodness-of-fit coefficient (gof) of the calibration curves, which were all <9.07%, the r, which were all >0.9940, and the limit of detection (LOD) and limit of quantification (LOQ).

#### 2.5.2. Accuracy and Precision

The results of the within- and between-day precision and accuracy evaluation are summarized in Table 3. For both the precision and accuracy, the acceptability ranges were met for all analytes at the specified levels.

#### 2.5.3. Specificity

In the chromatograms of blank porcine feces (Figure S2) no endogenous peaks were observed at the elution times of cefquinome, ceftiofur and DFCA, demonstrating the good specificity of the methods.

#### 2.5.4. Extraction Recovery (R_E_) and Signal Suppression and Enhancement (SSE)

The details of recovery and matrix effects of ceftiofur, cefquinome and their respective internal standards can be found in Table 4. Although the R_E_ of cefquinome and ceftiofur was limited, the extraction method still resulted in a LOD of 0.64 ng g^−1^ and 0.89 ng g^−1^, respectively. Porcine feces caused a matrix effect for both cefquinome (116.4%) and ceftiofur (266.8%). To minimize the influence of the extraction protocol and the matrix on the analytes, isotopically labelled internal standards were used, i.e., cefquinome-d7 and ceftiour-d3. Since the SSE and R*_E_* of both analytes are within the same range of their internal standards, the internal standards can account for the signal enhancement caused by the sample matrix.

## 3. Discussion

In literature, chromatographic separation of ceftiofur and cefquinome is mostly performed by using a C18 column [31,32,33,34,35,36,37]. A few methods use a PLRP-S column because of the robustness of this type of column in combination with an acidic mobile phase [38,39]. During method development, several C18 columns (Acquity BEH C18, 50 × 2.1 mm i.d., dp: 1.7 μm, Waters; PLRP-S, 150 × 2.1 mm i.d., dp: 5.0 μm, Agilent; Zorbax Eclipse plus C18, 100 × 3.0 mm i.d., dp: 3.5 µm, Agilent; Zorbax Eclipse plus C18, 100 × 2.1 mm i.d., dp: 3.5 µm), mobile phases (H_2_O, ACN and methanol alone and in combination with ammonium formate, FA, acetic acid) and gradient programs were evaluated. Signal intensity, retention time, matrix peaks and peak shape challenges were encountered. Finally, a Zorbax Eclipse plus C18 100 × 2.1 mm i.d. in combination with H_2_O containing 0.1% (*v*/*v*) FA and 2 mM ammonium formate as mobile phase A and ACN containing 0.1% (*v*/*v*) FA as mobile phase B was used for the cefquinome-ceftiofur method.

The results of the bench top stability experiments indicate that rapid degradation of ceftiofur and its metabolites occurs in feces at room temperature. This observation is in line with previously reported data by Hornish et al. [4]. To minimize the impact of the lag time between sampling and freezing on the measured concentration of the analytes, two different protocols were tested. The first protocol was snap-freezing 0.5 g of the sample. The second protocol was the addition of a β-lactamase inhibitor, tazobactam (500 µL, 4–8–16 µg mL^−1^). Addition of 500 µL of an 8 µg mL^−1^ solution of tazobactam in ultrapure water prior to storage at ≤−70 °C resulted in a recovery of at least 83% after 30 min at room temperature. This recovery falls within the accuracy ranges of −20 to +10%, and is therefore sufficient to bridge the sampling-storage lag time. Nonetheless, the time between sampling and freezing needs to be kept as short as possible. The freeze-thaw and extract stability did not meet the acceptability ranges for some analytes. Therefore, incurred samples have to be analyzed immediately on the day of extraction to avoid degradation prior to the quantification.

During method development, the focus was set on a straightforward and robust method. In the present study the extraction was developed in two steps. First, the best solvent to extract the analytes from fecal samples was selected. Several solvents (acetonitrile, formic acid, acetic acid and ultrapure water) at different ratios, mixtures and volumes were tested. Methanol was not used, since the use of methanol for extraction of β-lactams is not recommended due to degradation [40]. The highest extraction recovery was observed with 1.5 mL of a 1% (*v*/*v*) formic acid in acetonitrile-water (50:50, *v*/*v*) solution (results not shown). The second step was to purify the samples in order to improve column longevity. Adsorption solid phase extraction (SPE), ion exchange SPE, QuEChERS (Quick, Easy, Cheap, Effective, Rugged, and Safe) SPE and a combination of these sample clean-up techniques were tested. The best results were obtained with an Oasis PRiME HLB column (60 mg, 3 mL). These two steps were sufficient to clean up the samples for ceftiofur and cefquinome analysis. In order to obtain the total ceftiofur and ceftiofur metabolites concentration, this first clean-up was followed by a derivatization procedure. This procedure was adapted from a previously described method for the analysis of ceftiofur and its metabolites in horse plasma and synovial fluid [38]. Preliminary experiments were performed on spiked samples with various volumes of hydrolysis solution (6–15 mL) to determine the volume of dithioerythritol (DTE) solution that was needed to completely hydrolyze ceftiofur and its metabolites into desfuroylceftiofur. It was observed that 10 mL of the hydrolysis solution was sufficient. However, in order to reach an excess of DTE, a volume of 12 mL of the DTE solution per 0.5 g of feces was used in our final procedure. Similar preliminary experiments were conducted to determine the necessary volume of iodoacetamide solution (0.5–5 mL) needed to derivatize all desfuroylceftiofur into DFCA. Again, an excess of the solution (3.2 mL per 0.5 g feces) was used in our final procedure. Afterwards, the same solid-phase extraction clean-up as described by De Baere et al. [38] was used, showing good results. Finally, the extract was filtered through a PVDF (polyvinylidene fluoride) syringe filter for optimal clean-up prior to analysis. In both final methods, 500 µL of the 8 µg mL^−1^ tazobactam solution was added post sampling to prevent degradation. To avoid excessive dilution in the ceftiofur-cefquinome method, the volume of the 1% (*v*/*v*) formic acid in acetonitrile-water (50:50, *v*/*v*) solution was reduced to 1 mL. In the DFCA method, the final SPE extract was dried under nitrogen, therefore, adding 1.5 mL 1% (*v*/*v*) formic acid in acetonitrile-water (50:50, *v*/*v*) at the beginning of the extraction does not cause a dilution of the final sample.

The various validation criteria (i.e., linearity, accuracy, precision, LOQ, LOD and specificity) for the two methods were met. During the method development, the extraction recovery and signal suppression or enhancement of DFCA was not assessed. However, since the derivatization method derivatizes both the analyte and its isotopically labeled internal standard, ceftiofur-d3, and since a matrix-matched calibration curve is used, it can be assumed that these two measures compensate for matrix effects on the MS/MS system [41].

## 4. Materials and Methods

### 4.1. Blank Fecal Samples

Cefquinome, ceftiofur and ceftiofur metabolites-free feces was obtained from 8-week-old pigs of approximately 20 kg bodyweight (*n* = 6; Belgian landrace × Large white, Seghers Hybrid^®^, Wuustwezel, Belgium), which were not previously treated with ceftiofur or cefquinome. A mixture of both male and female feces was stored at ≤−15 °C for later use.

### 4.2. Standards and Chemicals

Ceftiofur, cefquinome sodium, dimethylsulfoxide (DMSO) and tazobactam were purchased from Sigma-Aldrich (Overijse, Belgium). Cefquinome-d7, ceftiofur-d3 and desfuroylceftiofur were purchased from Toronto Research Chemicals (North York, ON, Canada). Methanol and acetonitrile were of UHPLC grade and were obtained from Fisher Scientific (Erembodegem, Belgium). Water was obtained from a Milli-Q water purification system. Products and solvents used for preparation of buffer solutions and for extraction (acetic acid, formic acid or FA, potassium di-hydrogen phosphate or KH_2_PO_4_, potassium hydroxide or KOH, ammonium formate, potassium chloride or KCl, sodium-tetraborate, dithioerythritol or DTE, iodoacetamide) were obtained from Merck (Darmstadt, Germany) and Sigma-Aldrich (Overijse, Belgium).

#### 4.2.1. Preparation of Solutions

The hydrolysis solution for the DFCA analysis was prepared by dissolving 3.7 g of potassium chloride, 19.0 g sodium-tetraborate and 4.0 g of dithioerythritol in 1000 mL of ultrapure water. The hydrolysis solution was stored at 2–8 °C for a maximum of 5 days. The phosphate buffer (0.025M, pH 7.0) necessary for the iodoacetamide solution was prepared by adding 3.4 g of KH_2_PO_4_ to 700 mL of water and adjusting the pH to 7.0 with concentrated potassium hydroxide. Water was added to obtain a total volume of 1000 mL. Thereafter, 14 g of iodoacetamide was dissolved in 100 mL phosphate buffer to obtain a 14% (*w*/*v*) iodoacetamide solution. The iodoacetamide solution was stored at 2–8 °C and shielded from light. A 1 mg mL^−1^ stock solution of tazobactam was prepared in water. This stock solution was diluted with UHPLC-water to a concentration of 4, 8 and 16 µg mL^−1^ and stored at ≤−70 °C. A separate stock solution of 1 mg mL^−1^ of ceftiofur and ceftiofur-d3 was prepared in DMSO. The cefquinome stock solution of 1 mg mL^−1^ was prepared in UHPLC-water. Desfuroylceftiofur and cefquinome-d7 stock solutions of 1 mg mL^−1^ were prepared in methanol/DMSO 10/1 (*v*/*v*). The stock solutions were divided in Eppendorf cups (Novolab, Geraardsbergen, Belgium) and stored at ≤−70 °C. The stock solutions of the internal standards (IS, cefquinome-d7 and ceftiofur-d3 mix, and ceftiofur-d3) were diluted with UHPLC-water to a concentration of 25 µg mL^−1^. For the ceftiofur and cefquinome analysis, mixed working solutions of 10 µg mL^−1^, 1 µg mL^−1^ and 0.1 µg mL^−1^ were prepared. For the desfuroylceftiofuracetamide analysis, the stock solution of ceftiofur was diluted with UHPLC-water to obtain working solutions of 10 µg mL^−1^, 1 µg mL^−1^ and 0.1 µg mL^−1^

#### 4.2.2. Calibration Curve

Blank feces was spiked with the ceftiofur and cefquinome mixed working solutions to obtain calibrators with a concentration ranging from 5 ng g^−1^ to 1000 ng g^−1^ (5, 10, 25, 50, 100, 250, 500 and 1000 ng g^−1^). For the desfuroylceftiofuracetamide analysis, the concentration of the calibrators ranged from 30 ng g^−1^ to 2000 ng g^−1^ (30, 50, 100, 250, 500, 750, 1000 and 2000 ng g^−1^).

### 4.3. Fecal Analysis

#### 4.3.1. Determination of Ceftiofur and Cefquinome

##### Sampling Protocol

The sampling protocol was based upon the results of the bench-top stability studies (see Section 2.4.2). The samples were collected via rectal stimulation. From each fecal sample, several aliquots of 0.5 g were weighted and to each aliquot 500 µL of an 8 µg mL^−1^ tazobactam solution in water was added. Subsequently, the aliquots were vortexed and stored at ≤−70 °C within 30 min after sampling.

##### Extraction

To the frozen feces–tazobactam aliquot, 20 µL of the IS working solution of 25 µg mL^−1^ ceftiofur-d3/cefquinome-d7 mix was added. After vortex mixing for 15 s, 1 mL 1% (*v*/*v*) formic acid in acetonitrile–water (50:50, *v*/*v*) was added and the sample was shaken for 30 min using a rotary tumbler. After a 10-min centrifugation (2851× *g* at 4 °C), the supernatant was transferred to an Oasis PRiME HLB column (60 mg, 3 mL, Waters, Antwerp, Belgium) and processed with minimal vacuum. A 10 µL aliquot of the eluate was injected onto the HPLC-MS/MS system.

##### HPLC-MS/MS

The HPLC-MS/MS method for the analysis of ceftiofur and cefquinome was performed using a Quattro Ultima^®^ triple quadrupole mass spectrometer from Waters (Millford, MA, USA), with Masslynx software (version 4.0). The liquid chromatography system consisted of a Waters Alliance 2695 system (Antwerp, Belgium). Separation was achieved on a Zorbax Eclipse Plus column (Reversed Phase C18, 100 mm × 2.1 mm i.d., dp: 3.5 µm) combined with a guard column of the same type both from Agilent Technologies (Diegem, Belgium). The mobile phases for chromatographic separation consisted of 0.1% (*v*/*v*) formic acid and 2 mM ammonium formate in H_2_O (A) and 0.1% (*v*/*v*) formic acid in ACN (B). Flow rate was set at 400 µL min^−1^. Table 5 shows the gradient used for the liquid chromatographic separation of the analytes. Ten-microliter aliquots of the extracts were injected onto the HPLC-MS/MS-system. The temperature of the autosampler tray was set at 5.0 °C. The HPLC-MS/MS conditions for the ESI source used in positive ionization mode were optimized using direct infusion of working solutions, at 1 µg mL^−1^. The following tune parameters were obtained: capillary voltage, 3.5 kV; cone voltage, 20 V; source temperature, 120 °C; desolvation temperature, 400 °C. The optimal settings for collision energy for fragmentation of the molecular ion (or precursor ion), were 20 eV for ceftiofur and desfuroylceftiofur, and 15 eV for cefquinome.

Acquisition was performed in the selected reaction monitoring (SRM) mode. For cefquinome, cefquinome-d7, ceftiofur and ceftiofur-d3, the following transitions were followed (* quantification ion, most intense product ion): cefquinome mass-to-charge ratio (*m*/*z*) 529.0 > 134.0 *, 396.3; cefquinome-d7 *m*/*z* 536.4 > 141.0 *; ceftiofur: *m*/*z* 524.0 > 240.9 *, 126.0 and ceftiofur-d3: *m*/*z* 527.0 > 244.1 *.

#### 4.3.2. Determination of Desfuroylceftiofuracetamide

##### Sampling Protocol

The same sampling protocol as in Section 4.3.1.1 was used. The samples were collected via rectal stimulation. From each fecal sample, several aliquots of 0.5 g were weighted and to each aliquot 500 µL of an 8 µg mL^−1^ tazobactam solution in water was added. Subsequently, the aliquots were vortexed and stored at ≤−70 °C within 30 min after sampling.

##### Extraction

To the frozen feces–tazobactam aliquot, 20 µL of the working solution of 25 µg mL^−1^ ceftiofur-d3 was added. The sample was vortex mixed for 15 s, then 1.5 mL 1% (*v*/*v*) formic acid in acetonitrile–water (50:50, *v*/*v*) was added and the sample was vortexed for 15 s and then shaken for 30 min using a rotary tumbler. After 10-min centrifugation (2851× *g* at 4 °C), the supernatant was transferred onto an Oasis PRiME HLB column (60 mg, 3 mL) and processed with minimal vacuum. The eluate was transferred to a new tube and 12 mL of the DTE solution was added. The sample was then vortex mixed and placed in a water bath at 50 °C (±5 °C) for 15 min. Thereafter, the sample was allowed to cool down and 3.2 mL of the iodoacetamide solution was added. After thoroughly mixing the sample, it was shielded from light and left at room temperature for 30 min. Following derivatization, the sample was centrifuged for 10 min (2851× *g* at 4 °C). A 60 mg, 3 mL Oasis HLB SPE cartridge was conditioned using 1 mL of methanol followed by 1 mL of ultrapure water. The entire supernatant was applied onto the SPE cartridge, which was washed with 1 mL of 5% methanol in ultrapure water. After drying the cartridge under vacuum for 10 min, DFCA was eluted from the SPE cartridge using 1 mL of 5% acetic acid in acetonitrile. The eluent was evaporated to dryness (45 °C, N_2_) and the residue was re-dissolved in 150 µL of 0.005% (*v*/*v*) formic acid in ultrapure water, vortex mixed for 15 s, filtered through a PVDF syringe filter (0.22 µm pore size Millex-GV, Merck-Millipore, Overijse, Belgium) and transferred to an autosampler vial. A 5-microliter aliquot was injected onto the UPLC-MS/MS system.

##### UPLC-MS/MS Analysis

The UPLC-MS/MS analysis of DFCA was performed using a Quattro Premier XE^®^ triple quadrupole mass spectrometer from Micromass (Manchester, UK), with Masslynx software (version 4.0). The liquid chromatography system consisted of a Waters Acquity UPLC sample and solvent manager (Milford, MA, USA). Separation was achieved on a Acquity UPLC BEH C18 column (Reversed Phase C18, 50 mm × 2.1 mm i.d., dp: 1.7 µm) combined with a guard column of the same type, both from Waters. The mobile phases for chromatographic separation consisted of 0.005% (*v*/*v*) formic acid in H_2_O (A) and ACN (B). Flow rate was set at 300 µL min^−1^. Table 6 shows the gradient used for the liquid chromatographic separation of the analytes. Five µL aliquots of the extracts were injected onto the UPLC-MS/MS-system. The temperature of the autosampler tray was set at 10.0 °C. The MS/MS conditions for the ESI source used in positive ionization mode were based upon a previously in-house developed method for the detection of DFCA in muscle. The following tune parameters were used: capillary voltage, 2.5 kV; cone voltage, 35 V; source temperature, 120 °C; desolvation temperature, 250 °C. The optimal settings for collision energy for fragmentation of the molecular ion (or precursor ion), were 22 eV for DFCA and for DFCA-d3.

Acquisition was performed in the selected reaction monitoring (SRM) mode. For DFCA and DFCA-d3, the following transitions were followed (* quantification ion, most intense product ion): DFCA *m*/*z* 487.2 > 241.2 *, 167.1 and DFCA-d3 *m*/*z* 490.2 > 244.2 *.

#### 4.3.3. Analysis of Biological Samples

The applicability of the method for ceftiofur and cefquinome and the method for DFCA was evaluated by the analysis of fecal samples of two male healthy pigs (±18 kg body weight, Belgian landrace × Large white, Seghers Hybrid^®^, Wuustwezel, Belgium). The first male pig received a single IM dose of ceftiofur at 3 mg kg^−1^ body weight, in accordance with the leaflet (Excenel flow^®^, Zoetis, Louvain-la-Neuve, Belgium). The second pig received a single IM dose of cefquinome at 2 mg kg^−1^ body weight, in accordance with the leaflet (Ceffect^®^, Emdoka, Hoogstraten, Belgium). Different timepoints after administration (2, 4, 6, 8, 10, 12, 24 h) an aliquot of 0.5 g feces was sampled and 500 µL of an 8 µg mL^−1^ tazobactam solution in water was immediately added. The fecal samples were stored within 30 min post sampling at ≤−70 °C until analysis. The housing conditions were in accordance with Belgian law (Royal Decree of the 17th of February 2017 “KB on the protection of experimental animals” [42]) and the pigs had ad libitum access to water and food.

The animal experiment was approved by the Ethical Committee of the Faculty of Veterinary Medicine and the Faculty of Bioscience Engineering of Ghent University (approval number EC 2021–16).

### 4.4. Stability Experiments

#### 4.4.1. Freeze-Thaw, Matrix and Extract Storage Stability

The freeze-thaw stability of cefquinome and ceftiofur, and of the main metabolite desfuroylceftiofur was verified by analyzing three independent samples for each detection method after three cycles of freezing and thawing on three consecutive days. These samples were spiked at different concentration levels (1000 ng g^−1^, 100 ng g^−1^ and 5 ng g^−1^ ceftiofur/cefquinome; 2000 ng g^−1^, 500 ng g^−1^ and 30 ng g^−1^ ceftiofur for the derivatization method and 2000 ng g^−1^, 500 ng g^−1^ and 30 ng g^−1^ desfuroylceftiofur), 500 µL of an 8 µg mL^−1^ tazobactam solution was added and after vortex mixing, the samples were stored at ≤−70 °C.

The storage stability in matrix of cefquinome, ceftiofur and desfuroylceftiofur was tested by analyzing six independent samples for each detection method at the same concentration levels as for the freeze-thaw stability test. To the spiked matrix samples, 500 µL of an 8 µg mL^−1^ tazobactam solution was added and after vortex mixing these samples were stored for 4 weeks at ≤−70 °C prior to analysis.

The storage stability in extract of cefquinome, ceftiofur and DFCA was verified by analyzing three extracted independent samples for each detection method at the same concentration levels as before. For the ceftiofur-cefquinome method, the liquid eluate was stored, whilst for the DFCA method the dried eluate was stored, both for 3 days at ≤−70 °C prior to analysis.

#### 4.4.2. Bench Top Stability

Stability at room temperature (21 ± 2 °C) of the ceftiofur working solution DMSO/UHPLC-water (1/1000; *v*/*v*) and cefquinome solution in UHPLC-water was tested by measuring the absolute chromatographic peak area in triplicate of a 1 µg mL^−1^ solution at 0, 0.5, 1, 1.5, 2 and 4 h after preparation of the working solution.

Bench top stability of ceftiofur, desfuroylceftiofur and cefquinome in matrix at room temperature (21 ± 2 °C) was tested by spiking eighteen independent samples of 0.5 g blank feces at a concentration level of 1000 ng g^−1^ ceftiofur/cefquinome for the ceftiofur/cefquinome method and by spiking eighteen samples of 0.5 g of blank feces at a concentration level of 1000 ng g^−1^ desfuroylceftiofur for the derivatization method. Three samples per method and per analyte were extracted and analyzed 0, 0.5, 1, 1.5, 2 and 4 h after spiking.

The effect of snap-freezing the samples on the stability of ceftiofur was also tested. Nine independent samples of 0.5 g of blank feces were spiked at a concentration level of 1000 ng g^−1^ ceftiofur. Three of them were extracted and analyzed instantly via the derivatization method. Three of them were snap-frozen in liquid nitrogen for 20 min, thawed for 20 min, extracted and analyzed. The last three were snap-frozen in liquid nitrogen for 20 min and extracted without thawing.

Besides snap-freezing, the addition of tazobactam, a β-lactamase inhibitor, on the stability of the analytes was also tested. The same protocol as for the tazobactam-free feces was used to determine the effect of the addition of 500 µL of a 4, 8 or 16 µg mL^−1^ solution of tazobactam in water on the stability of the analytes in matrix at room temperature (21 ± 2 °C) at different timepoints.

### 4.5. Validation Criteria

The (U)HPLC-MS/MS methods for the quantification of cefquinome, ceftiofur and DFCA were in-house validated according to the Veterinary International Conference of Harmonization (VICH, guideline 49) [43] and EU recommendations [44,45,46].

#### 4.5.1. Linearity

The linearity was determined on calibration curves using spiked blank fecal samples (for levels, see Section 4.2.2). Peak area ratios of cefquinome, ceftiofur, DFCA and their respective IS were plotted against their concentration and a linear regression was performed using a 1/x**^2^** fit weighting for cefquinome and ceftiofur and a quadratic regression was performed using a 1/x**^2^** fit weighting for DFCA. For each calibration curve, the correlation coefficient (r) and the goodness of fit (gof), i.e., how well the calibration curves fit the regression models, were determined and should fall within the ranges specified (r ≥ 0.99, gof ≤ 10%) [45]. The gof (%) was calculated according to the formula below.

$$g=\sqrt{\Sigma {\left(\%\;deviation\right)}^{2}/\left(n-1\right)}$$

with % deviation = x_calculated conc_ − x_nominal value/_x_nonimal value_ ×100.

#### 4.5.2. Accuracy

The accuracy was determined by evaluating six independently spiked blank fecal samples at concentration levels of 1000 ng g^−1^, 100 ng g^−1^ and 5 ng g^−1^ ceftiofur/cefquinome and 2000 ng g^−1^, 500 ng g^−1^ and 30 ng g^−1^ ceftiofur for the derivatization method. The within-day accuracy (*n* = 6) and between-day accuracy (three different days, *n* = 18), expressed as the difference between the mean found concentration and the theoretical concentration (in %), had to be within −20 to +10%.

The accuracy of the samples of the freeze-thaw, matrix and extract stability experiments (see Section 4.4.1) also had to fall within these margins.

#### 4.5.3. Precision

The precision was expressed as the relative standard deviation (RSD) (in %), which is the ratio between the standard deviation (SD) and the mean found concentration. The RSD (%) had to fall within the ranges listed in the VICH GL49 [43]. The latter values were calculated according to the Horwitz equation: RSD = 2^(1**−**0.5*log c)^, with c the analyte concentration expressed as a dimensionless mass fraction. The same samples as used for the accuracy evaluation were used to determine the precision.

The within-day precision was evaluated by analyzing six independently spiked blank fecal samples at concentration levels of 1000 ng g^−1^, 100 ng g^−1^ and 5 ng g^−1^ ceftiofur/cefquinome and 2000 ng g^−1^, 500 ng g^−1^ and 30 ng g^−1^ ceftiofur for the derivatization method. The between-day precision was evaluated by analyzing three times six samples at each concentration level, prepared and analyzed on three different days.

The precision of the samples of the freeze-thaw, matrix and extract stability experiments (see Section 4.4.1) also had to fall within the ranges listed in the VICH GL49 [43].

#### 4.5.4. Limit of Quantification

The LOQ is defined as the lowest concentration for which the method is validated with an accuracy and a precision that fall within the ranges (mentioned in the section accuracy and precision). The LOQ was determined by analyzing six spiked samples on the same day. Furthermore, the LOQ was the lowest point of the calibration curve.

#### 4.5.5. Limit of Detection

The LOD is defined as the lowest concentration that could be determined with a signal-to-noise (S/N) ratio of ≥3. The LOD was determined by calculating the analyte concentration that corresponds to a S/N ratio of 3/1 based on the S/N ratio of the quantification ion of the analyte in six LOQ samples.

#### 4.5.6. Specificity

Specificity was evaluated by extracting and analyzing blank fecal samples with the abovementioned methods. The area of the endogenous peak at the retention time of the analytes had to be ≤20% than the average area of the LOQ of that analyte. For the internal standards, the area of the endogenous peak had to be ≤5% than the average area of the internal standard peak.

#### 4.5.7. Extraction Recovery (R_E_) and Signal Suppression and Enhancement (SSE)

To assess the extraction recovery and signal suppression and enhancement of ceftiofur and cefquinome, three batches of samples were prepared. These batches consisted of three independent samples at three concentration levels (1000 ng g^−1^, 100 ng g^−1^ and 5 ng g^−1^ ceftiofur/cefquinome). The first batch (A) consisted of blank matrix samples spiked with the two analytes before extraction. The second batch (B) consisted of blank matrix samples. After extraction of these blank samples, the same volume of working solutions as in batch A was added to the extract. The final batch (C) was prepared by adding the same volume of working solutions as in batch A to 500 µL of the 8 µg mL^−1^ tazobactam solution and adding 1 mL 1% formic acid in acetonitrile-water (50:50, *v*/*v*). R_E_ and SSE were determined by dividing the peak areas of the analytes, using the following formulas:

$$\mathrm{SSE}\;\left(\%\right)=\frac{B}{C}\times 100$$

$$R_{E}\;\left(\%\right)=\frac{A}{B}\times 100$$

## 5. Conclusions

The aim of this study was to develop and validate (U)HPLC–MS/MS methods for the quantitative determination of cefquinome, ceftiofur and ceftiofur-related metabolites in porcine feces. During method development, emphasis was placed on the matrix stability of the analytes. To obtain reliable results, it was imperative to add a β-lactamase inhibitor to prevent degradation prior to analysis. The validation results for the different parameters fell within the specified ranges. Next, these methods can be applied to pharmacokinetic studies, and to determine the possible effect of parenteral administration of cefquinome and ceftiofur on the exposure of gut bacteria.

## Figures and Tables

**Table 1:** Liquid chromatography-tandem mass spectrometric parameters for the analysis of cefquinome (CQ), ceftiofur (CT) and desfuroylceftiofuracetamide (DFCA) and their respective internal standards (CQ-d7, CT-d3 and DFCA-d3) in spiked porcine feces.

Analyte	Retention Time (min)	Precursor Ion (*m*/*z*)	Cone Voltage (V)	Product Ions (*m*/*z*) (Collision Energy eV)	
				Quantifier	Qualifier
CQ	4.91	529.0	40	134.0 (15)	396.3 (15)
CQ-d7	4.90	536.4	15	141.0 (20)	
CT	8.35	524.0	70	240.9 (20)	126.0 (20)
CT-d3	8.34	527.0	60	244.1 (20)	
DFCA	1.96	487.2	35	241.2 (22)	167.1 (22)
DFCA-d3	1.93	490.2	35	244.2 (22)	

**Table 2:** Results of the evaluation of linearity (goodness-of-fit coefficient (gof), correlation coefficient (r), mean ± standard deviation), limit of detection (LOD), and limit of quantification (LOQ) of cefquinome (CQ), ceftiofur (CT) and desfuroylceftiofuracetamide (DFCA) in porcine feces.

Analyte	Calibration Range (ng g^−1^)	Gof ^a^ (%) (*n* = 6)	R ^a^ (*n* = 6)	LOD (ng g^−1^)	LOQ (ng g^−1^)
CQ	5–1000	3.70 ± 1.43	0.9990 ± 0.0007	0.64	5
CT	5–1000	5.51 ± 1.14	0.9979 ± 0.0009	0.89	5
DFCA	30–2000	7.31 ± 1.76	0.9960 ± 0.0020	1.47	30

^a^ Acceptance criteria: r > 0.99 and gof < 10%.

**Table 3:** The within-day (*n* = 6) and between-day (*n* = 18) precision and accuracy data for cefquinome (CQ), ceftiofur (CT) and desfuroylceftiofuracetamide (DFCA).

Analyte	Target Concentration (ng g^−1^)	Mean Concentration ± SD (*n* = 18) (ng g^−1^)	RSD (%) Within-Day (*n* = 6) /Between-Day (*n* = 18)	Accuracy (%) Within-Day (*n* = 6) /Between-Day (*n* = 18)
CQ	5	4.7 ± 0.3	6.9/6.5	−8.4/−6.2
	100	91.8 ± 4.5	3.1/4.9	−6.0/−8.2
	1000	939.0 ± 37.8	2.8/4.0	−3.0/−6.1
CT	5	5.1 ± 0.3	4.5/5.0	−3.5/+1.1
	100	101.1 ± 2.6	3.1/2.6	+0.7/+1.1
	1000	1047.4 ± 31.9	4.1/3.0	+3.9/+4.7
DFCA	30	29.4 ± 2.2	7.8/7.4	−4.6/−2.0
	500	480.6 ± 47.5	7.6/9.9	−10.7/−3.9
	2000	1952.6 ± 164.3	9.3/8.4	0.3/−2.4

**Table 4:** Signal suppression and enhancement (SSE) and extraction recovery (R_E_) of cefquinome, ceftiofur and their internal standards, cefquinome-d7 and ceftiofur-d3, in porcine feces.

Analyte	SSE (%)	R_E_ (%)
Cefquinome	116.4 ± 13.4	41.2 ± 3.1
Cefquinome-d7	119.2 ± 11.3	42.6 ± 4.4
Ceftiofur	266.8 ± 47.8	25.8 ± 3.0
Ceftiofur-d3	233.2 ± 17.2	28.3 ± 3.1

**Table 5:** Gradient used for the separation of cefquinome, cefquinome-d7, ceftiofur and ceftiofur-d3. With mobile phase A: 0.1% (*v*/*v*) formic acid and 2mM ammonium formate in H_2_O and mobile phase B: 0.1% (*v*/*v*) formic acid in acetonitrile.

Time (min)	Mobile Phase A (%)	Mobile Phase B (%)
0–1.0	95	5
1.0–5.0	95–60	5–40
5.0–8.0	60–5	40–95
8.0–10.0	5	95
10.0–10.1	5–95	95–5
10.1–15.0	95	5

**Table 6:** Gradient used for the analysis of desfuroylceftiofuracetamide and desfuroylceftiofuracetamide-d3. With mobile phase A: 0.005% (*v*/*v*) formic acid in H_2_O and mobile phase B: acetonitrile.

**Time (min)**	**Mobile Phase A (%)**	**Mobile Phase B (%)**
0–4.0	91	9
4.0–4.1	91–10	9–90
4.1–4.6	10–0	90–100
4.6–8.6	0	100
8.6–8.7	0–91	100–9
8.7–12.0	91	9

## Supplementary Materials

The following are available online. Figure S1: Chemical structures, MS and MS/MS spectra, Figure S2: MS/MS chromatograms Table S1: Freeze-thaw, matrix and extract stability results.

## Sample Availability

Incurred fecal samples of the compounds are available from the authors.
