# Supplementary material for: Development and Validation of Liquid Chromatography-Tandem Mass Spectrometry Methods for the Quantification of Cefquinome, Ceftiofur, and Desfuroylceftiofuracetamide in Porcine Feces with Emphasis on Analyte Stability
Source: Molecules. 2021 Jul 29;26(15):4598. doi: 10.3390/molecules26154598 (PMC8348739; doi:10.3390/molecules26154598)
Supplement: Supplementary file 1 [file molecules-26-04598-s001.zip › molecules-1272865-supplementary.pdf]

Supplementary data

Development and validation of liquid chromatography-tandem mass spectrometry methods for the quantification of cefquinome, ceftiofur, and desfuroylceftiofuracetamide in porcine feces with emphasis on analyte stability

Sofie Rutjens [1], Siska Croubels [1], Siegrid De Baere [1] and Mathias Devreese [1,*]

[1] Department of Pharmacology, Toxicology and Biochemistry, Faculty of Veterinary Medicine, Ghent University, Merelbeke, Belgium; Sofie.Rutjens@UGent.be
* Corresponding Author: Mathias Devreese e-mail: Mathias.Devreese@UGent.be tel: 32 (0)92647347

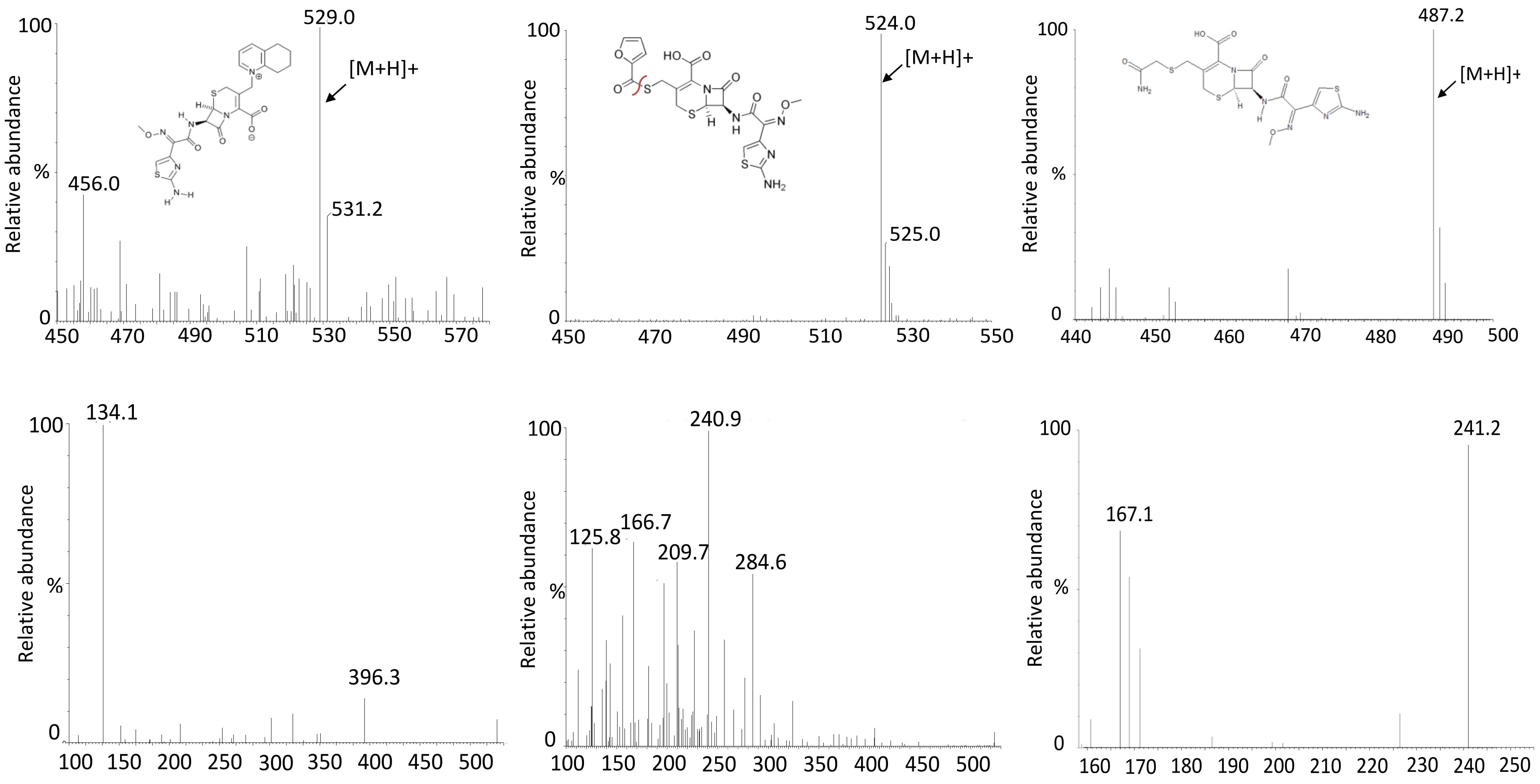

Figure S1. Chemical structure, MS and MS/MS spectra of cefquinome, ceftiofur and desfuroylceftiofuracetamide obtained after direct infusion of working solutions of 1 μg mL^{-1} of cefquinome and ceftiofur and the direct infusion of a derivatized ceftiofur working solution of 1 μg mL^{-1} for detection of desfuroylceftiofuracetamide (ESI positive mode, collision energy in MS/MS = 15, 20, 22 eV for cefquinome, ceftiofur and desfuroylceftiofuracetamide, respectively).

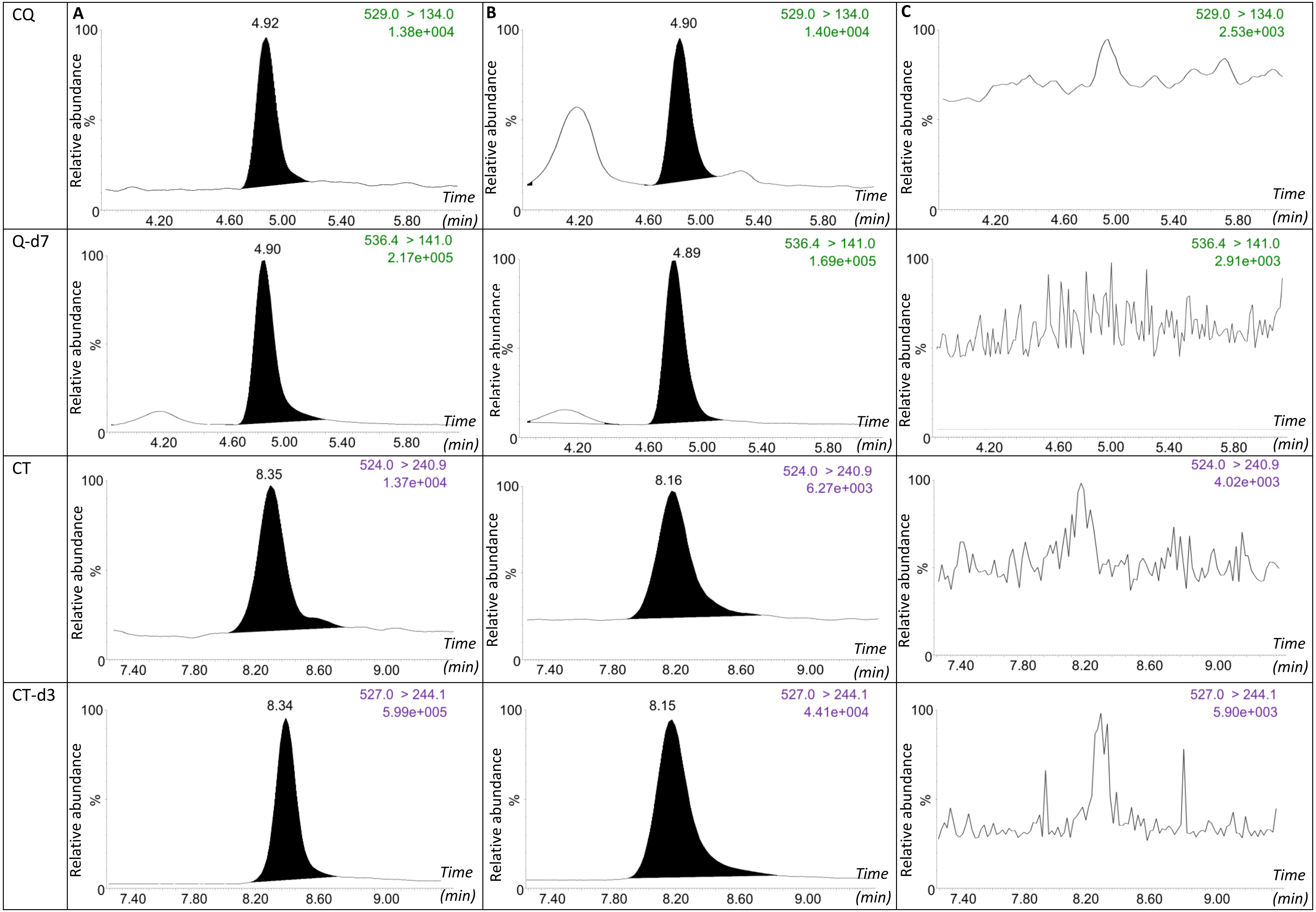
CQ
Q-d7
CT
CT-d3
A
B
C
4.92
4.90
529.0 > 134.0
1.38e+004
1.40e+004
2.53e+003
536.4 > 141.0
2.17e+005
1.69e+005
2.91e+003
4.89
8.35
8.16
524.0 > 240.9
1.37e+004
6.27e+003
4.02e+003
8.34
8.15
527.0 > 244.1
5.99e+005
4.41e+004
5.90e+003
Relative abundance
%
100
0
Time
(min)
4.20
4.60
5.00
5.40
5.80
7.40
7.80
8.20
8.60
9.00

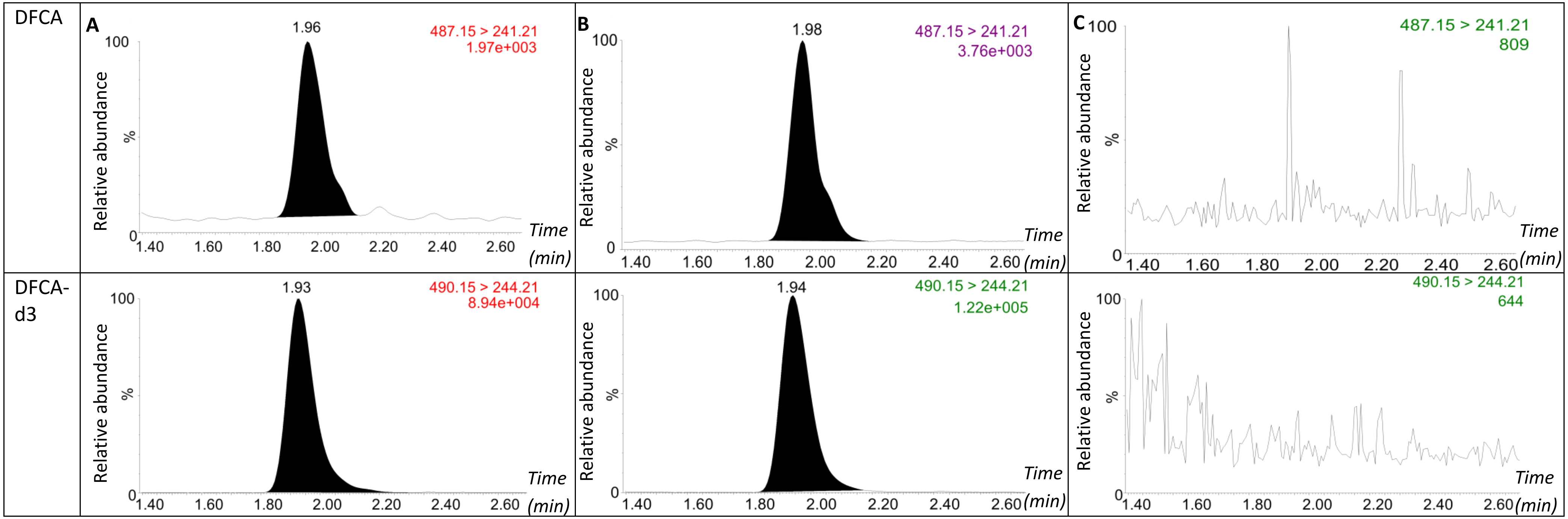

Figure S2 – MS/MS chromatograms of cefquinome (529 > 134), cefquinome-d7 (536.4 > 141), ceftiofur (524 > 240.9), ceftiofur-d3 (527 > 244.1), DFCA (487.2 > 241.2) and DFCA-d3 (490.2 > 244.2), for (A) blank pig fecal sample spiked at 5 ng g^{-1} for ceftiofur (CT) and cefquinome (CQ) and 30 ng g^{-1} for desfuroylceftiofuracetamide (DFCA) (Limit Of Quantification), of (B) incurred pig fecal sample (CQ, CT and DFCA concentration: 7.1 ng g^{-1}, 85.4 ng g^{-1} and 30.5 ng g^{-1}, respectively) and of (C) blank pig fecal sample.

Table S1. Freeze-thaw stability (n=3) after three cycles of freezing at ≤ -70 °C and thawing at room temperature, four weeks matrix stability (n=6) and three days extract stability (n=6), expressed as precision and accuracy data for cefquinome (CQ), ceftiofur (CT) and ceftiofur, measured as desfuroylceftiofuracetamide (DFCA). Freeze-thaw stability (n=3) after three cycles of freezing at ≤ -70 °C and four weeks matrix stability (n=6) precision and accuracy data for the main metabolite, desfuroylceftiofur (DFC), measured as desfuroylceftiofuracetamide.

**Analyte**		**Freeze-thaw stability (n=3)**			**Matrix stability (n=6) 4 weeks**			**Extract stability (n=6) 3 days**		
	Target concentration (ng g^{-1})	Mean concentration (ng g^{-1})	RSD (%)	Accuracy (%)	Mean concentration (ng g^{-1})	RSD (%)	Accuracy (%)	Mean concentration (ng g^{-1})	RSD (%)	Accuracy (%)
CQ	5	1.5 ± 0.2*	14.5	-67.3	5.0 ± 0.4	8.4	-1.0	4.7 ± 0.3	6.2	-5.6
	100	85.8 ± 4.2	4.8	-14.2	89.5 ± 7.8	8.7	-10.5	103.1 ± 2.9	2.8	+3.1
	1000	942.3 ± 58.9	6.3	-5.8	925.9 ± 72.1	7.8	-7.4	1062.0 ± 15.3	1.4	+6.2
CT	5	0.0 ± 0.0*	-	-100	5.0 ± 0.3	5.5	+0.2	4.2 ± 0.2	4.2	-15.1
	100	81.8 ± 0.5	0.6	-18.2	90.0 ± 3.0	3.3	-10.0	88.7 ± 2.3	2.6	-11.3
	1000	1064.9 ± 18.0	1.7	6.5	1065.3 ± 11.1	1.0	+6.5	943.5 ± 3.9	0.4	-5.7
DFCA[a]	30	29.4 ± 2.3	7.8	-2.1	31.3 1.8	5.8	+4.5	35.0 ± 1.8*	5.0	+16.8
	500	483.3 ± 11.3	2.3	-3.3	522.4 ± 12.2	2.3	+4.5	519.7 ± 11.9	2.3	+3.9
	2000	1985.8 ± 16.9	0.9	-0.7	2099.4 ± 61.2	2.9	+5	2102.1 ± 29.4	1.4	+5.1
DFC[b]	30	32.1 ± 4.2	13.2	+7.1	32.5 ± 4.7	14.4	+8.5			
	500	475.9 ± 34.6	7.3	-4.8	480.6 ± 35.9	7.5	-3.9			
	2000	2054.9 ± 92.0	4.5	+2.7	1850.8 ± 103.2	5.6	-7.5			

*Acceptability ranges were not met (RSD according to VICH GL49 [20], Accuracy between -20 to +10%)
[a] Derivatized samples spiked with ceftiofur
[b] Derivatized samples spiked with desfuroylceftiofur
